# Intraoperative dexmedetomidine and postoperative cerebral hyperperfusion syndrome in patients who underwent superficial temporal artery-middle cerebral artery anastomosis for moyamoya disease: A retrospective observational study

**DOI:** 10.1097/MD.0000000000005712

**Published:** 2016-12-30

**Authors:** Hyungseok Seo, Ho-Geol Ryu, Je Do Son, Jeong-Soo Kim, Eun Jin Ha, Jeong-Eun Kim, Hee-Pyoung Park

**Affiliations:** a Department of Anesthesiology and Pain Medicine, Dankook University Hospital, Cheonan; b Department of Anesthesiology and Pain Medicine; c Department of Neurosurgery, Seoul National University Hospital, Seoul National University College of Medicine, Seoul, Korea.

**Keywords:** cerebral hyperperfusion syndrome, dexmedetomidine, direct revascularization surgery, moyamoya disease

## Abstract

Dexmedetomidine, a selective α_2_-agonist, reduces cerebral blood flow and has neuroprotective effects against cerebral ischemia/reperfusion injury in experimental animals. We examined whether intraoperative dexmedetomidine would reduce the incidence of postoperative cerebral hyperperfusion syndrome (CHS) after superficial temporal artery-middle cerebral artery (STA-MCA) anastomosis in patients with moyamoya disease.

The electronic medical records of 117 moyamoya patients who underwent STA-MCA anastomosis were reviewed retrospectively. The patients were divided into 2 groups: 48 patients received intraoperative dexmedetomidine (Group D), while 69 patients did not (Group ND). The incidence (primary outcome), onset, and duration of postoperative CHS were noted.

The incidence of postoperative CHS was 45.8% and 40.6% in groups D and ND, respectively (*P* = 0.708). The duration of postoperative CHS was shorter in group D than in group ND (median [Q1–Q3], 5 [3–7] vs 8 [5–10] days, *P* = 0.021). There was no significant difference in the onset of CHS between group D and group ND (0 [0–2] vs 1 [0–3] days, *P* = 0.226).

In conclusion, intraoperative dexmedetomidine did not reduce the incidence of postoperative CHS, although it reduced the duration of CHS, in patients who had undergone direct revascularization surgery for moyamoya disease.

## 1 Introduction

In patients with moyamoya disease, a standard surgical therapeutic option is direct revascularization surgery, such as superficial temporal artery-middle cerebral artery (STA-MCA) anastomosis.^[1]^ Direct revascularization surgery can increase cerebral perfusion, thereby reducing the potential risk for cerebral ischemia. However, there is a potential risk for neurological complications due to cerebral hyperperfusion, which is called cerebral hyperperfusion syndrome (CHS) and it has been reported to occur in up to 50% of patients after STA-MCA anastomosis.^[1–3]^ Strict perioperative blood pressure control can help prevent CHS after direct revascularization surgery.^[4]^ However, there is no established optimal treatment strategy for the prevention and treatment of postoperative CHS.^[1]^

Dexmedetomidine, a selective α_2_-agonist, can reduce cerebral blood flow (CBF) and has neuroprotective effects against cerebral ischemia/reperfusion injury in experimental animals.^[4–6]^ It has been shown to be effective for the management of patients with postoperative CHS.^[7]^ However, to the best of our knowledge, no study has examined the preventive effects of intraoperative dexmedetomidine on postoperative CHS in patients who have undergone direct revascularization surgery for moyamoya disease.

We reviewed the medical records of patients who underwent STA-MCA anastomosis and investigated the effects of intraoperative dexmedetomidine on the incidence of postoperative CHS. We hypothesized that intraoperative dexmedetomidine would reduce the incidence of postoperative CHS.

## 2 Materials and methods

### 2.1 Patients

The Institutional Review Board of Seoul National University Hospital approved this study (number: 1506-069-680), and written informed consent was waived because of its retrospective nature. We retrospectively reviewed the electronic medical records of 117 moyamoya patients who underwent STA-MCA anastomosis from May 2012 to April 2015 at Seoul National University Hospital. Of these, 48 patients received intraoperative dexmedetomidine (Group D), while 69 patients did not (Group ND).

### 2.2 Anesthesia protocol

General anesthesia was induced and maintained with a continuous infusion of propofol (effect site concentration 3–6 μg/mL) and remifentanil (effect site concentration 3–6 ng/mL) using a target-controlled infusion pump (Orchaestra, Fresenius, Bad Homberg, Germany). The intraoperative mean arterial pressure (MAP) was strictly maintained at the level of the highest preoperative MAP ± 20 mmHg until the STA-MCA anastomosis was finished. If necessary, intravenous phenylephrine (20–30 μg) was administered intermittently or infused continuously at a rate of 10 to 20 μg/kg/h. After completing the STA-MCA anastomosis, dexmedetomidine infusion was initiated; a loading dose of dexmedetomidine 1.0 μg/kg over 15 minutes, followed by a continuous infusion at a rate of 0.3 to 0.5 μg/kg/h. During dexmedetomidine infusion, the MAP was maintained at the level of the lowest preoperative MAP ± 20 mmHg by changing effect site concentrations of propofol and remifentanil. Dexmedetomidine infusion was terminated before transfer to intensive care unit (ICU) and was not continued to the postoperative period in ICU. Hyperventilation was avoided to maintain the partial pressure of arterial carbon dioxide (PaCO_2_) at 35 to 40 mmHg during the surgery. The intraoperative hemoglobin concentration was maintained at a minimum level of 10 g/dL. All surgery was performed by 1 neurosurgeon. The surgical techniques, such as the craniotomy size, STA preparation and site (fourth branch of the MCA), and size of the anastomosis, were not changed during this study period.

### 2.3 Postoperative management

In all patients, the brain computed tomography (CT) examination was routinely performed at immediate postoperative period to detect surgery-related complications such as hematoma or infarction. All patients underwent complete neurological examination by neurosurgeons when they became fully awake. MAP was strictly maintained at the level of the lowest preoperative MAP ± 20 mmHg for about 3 days of postoperative period. If necessary, intravenous nicardipine (0.5–1 mg) was administered intermittently or infused continuously at a rate of 5 to 15 mg/h. Cerebral conventional angiography or magnetic resonance angiography was done on the seventh postoperative day to evaluate the patency of STA-MCA anastomosis and perfused area of the bypass in a few patients. If patients manifested neurologic symptoms and signs in the postoperative period, brain CT and diffusion magnetic resonance imaging (MRI) with arterial spin labeling were taken to evaluate postoperative changes in cerebral perfusion. If necessary, brain single-photon emission CT (SPECT) was performed in some cases to confirm the diagnosis of CHS.

### 2.4 Outcome measurement

We analyzed the presence of CHS and the timing of its occurrence and duration after STA-MCA anastomosis. As described previously,^[2]^ CHS was deemed present when all of the following criteria were met: new development of postoperative focal neurological deficits (for example, hand and tongue motor dysfunction and dysphasia), seizure, and symptomatic subarachnoid hemorrhage, which were not seen before the operation or in the immediate postoperative period; reversible postoperative neurological deficits that resolved completely within 15 days after the operation; no definite hematomas or acute infarction on brain CT and diffusion MRI; a significant focal increase in CBF at the site of the anastomosis on postoperative arterial spin labeling MRI and/or brain SPECT. Finally, a neurosurgeon, who was blinded to this study, confirmed that the patients had postoperative CHS.

The perioperative patient data consisted of the pre-, intra-, and postoperative factors. Preoperative factors were patient characteristics, initial clinical manifestations, hemodynamic variables at the general ward, the angiographic stage based on angiographic criteria, significant decreased perfusion on SPECT (CBF <50%), and laboratory findings. Intraoperative factors were the operating and anesthesia times, the operative side, fluid balance, hemodynamic variables, PaCO_2_, transfusion, and laboratory findings. Postoperative factors were the blood pressure on admission to the ICU, Acute Physiology and Chronic Health Evaluation II (APACHE II) score, and laboratory findings. The hospital course data included the hospital mortality and lengths of ICU and hospital stay.

### 2.5 Statistical analysis

Statistical analysis was performed using SPSS 22.0 (IBM SPSS Statistics for Windows, IBM Corp, Armonk, NY). The data were screened for normality with the Kolmogorov–Smirnov test. Continuous variables were compared using Student *t* test or the Mann–Whitney *U* test. Categorical data were analyzed using the *χ*^*2*^ or Fisher exact test. To determine independent risk factors for postoperative CHS, univariate analyses of the pre-, intra-, and postoperative data were performed and only variables with *P* values less than 0.20 were entered into a binary logistic regression with the forward stepwise conditional method. A *P* value <0.05 was considered statistically significant.

## 3 Results

Of the 117 patients who underwent STA-MCA bypass surgery, intraoperative dexmedetomidine was administered in 48 (41.0%) patients. Table 1 shows the patient demographics and preoperative data. No significant differences were found between the 2 groups.

**Table 1:**
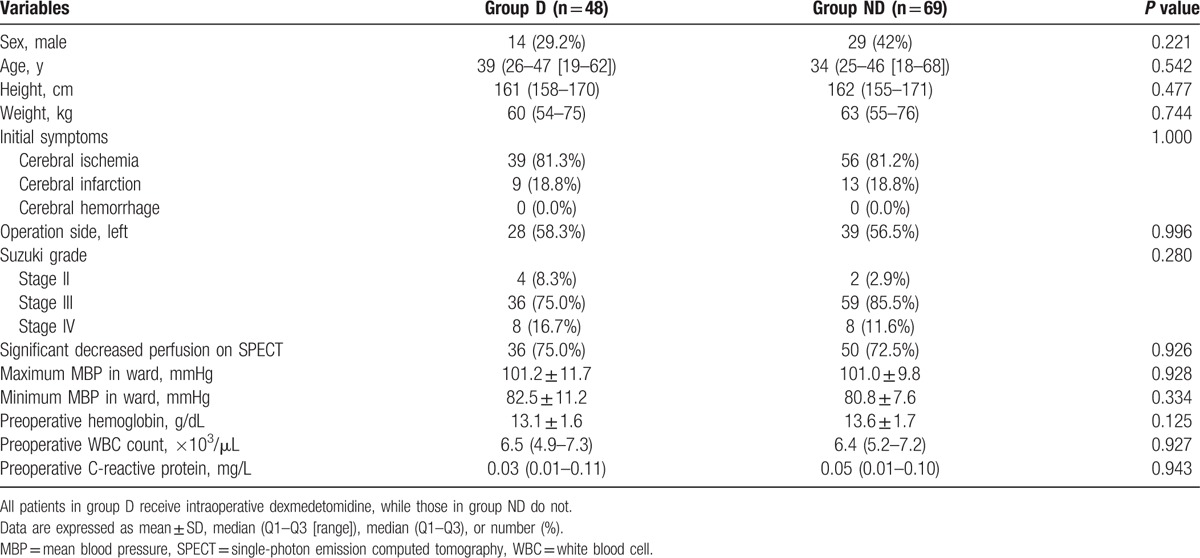
Demographics and preoperative data.

Table 2 summarizes the intra- and postoperative data. The median [Q1–Q3] value of urine output was higher in group D than in group ND (2085 [1610–2518] vs 1900 [1530–2100] mL, *P* = 0.045), while the MBP at the time of ICU admission was lower in group D (81.3 ± 16.1 vs 97.2 ± 16.8 mmHg, *P* < 0.001).

**Table 2:**
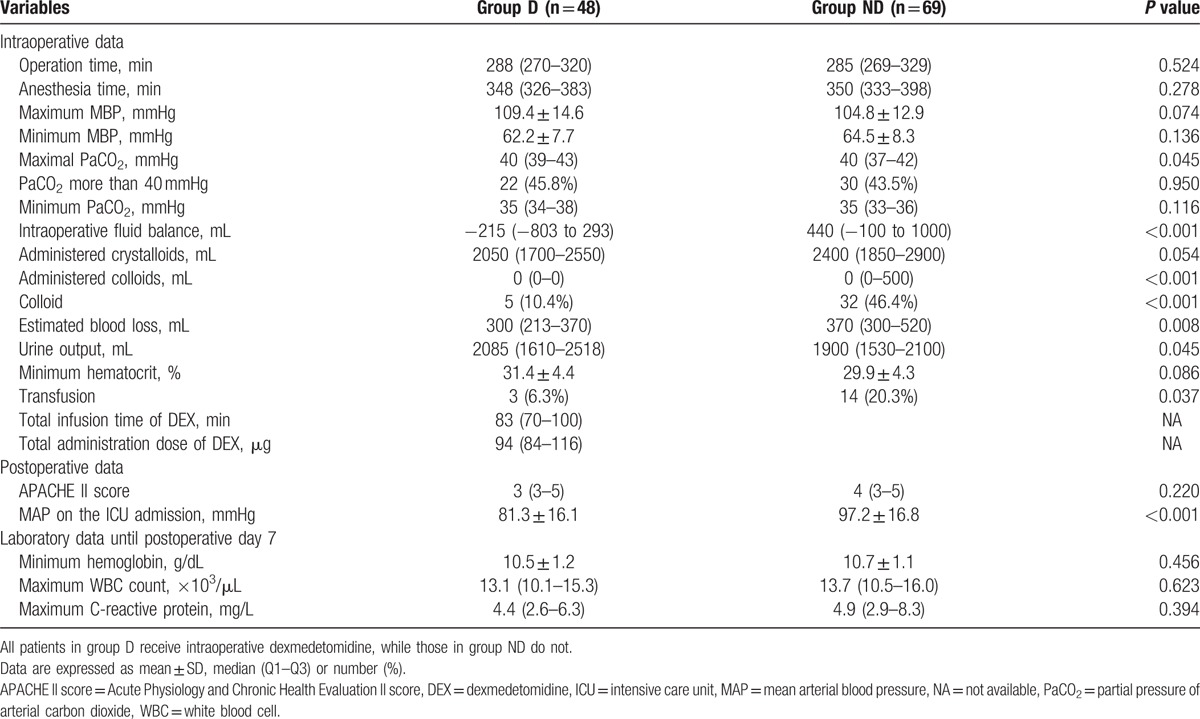
Intraoperative and postoperative data between 2 groups.

The incidences of postoperative CHS were 22/48 (45.8%) in group D and 28/69 (40.6%) in group ND (*P* = 0.708, Table 3). The duration of postoperative CHS was shorter in group D compared with group ND (5 [3–7] vs 8 [5–10] days, *P* = 0.021). The hospital stay was also shorter in group D (9 [8–10] vs 10 [9–12] days, *P* = 0.001).

**Table 3:**
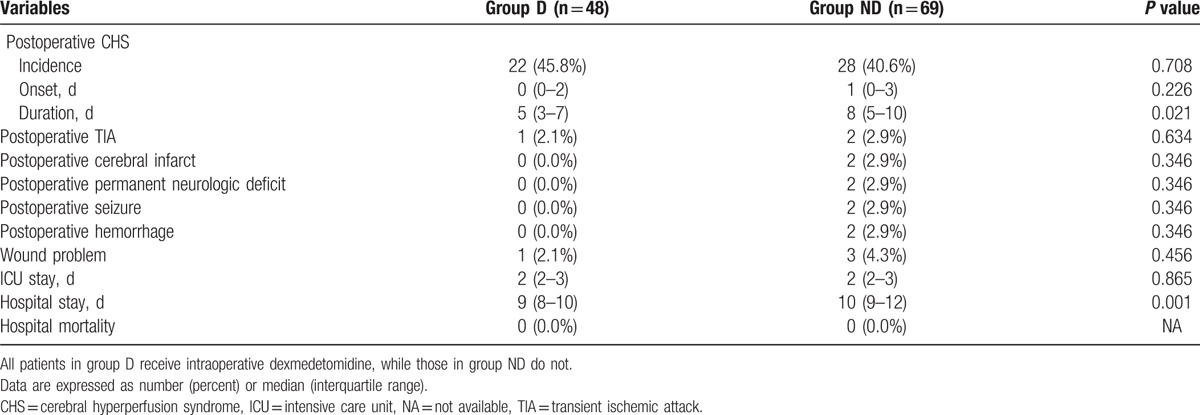
Hospital course between 2 groups.

On multivariate logistic analysis, an operation on the dominant hemisphere, female gender, and minimum MBP in ward were significantly associated with the development of postoperative CHS after STA-MCA anastomosis in patients with moyamoya disease (Table 4).

**Table 4:**
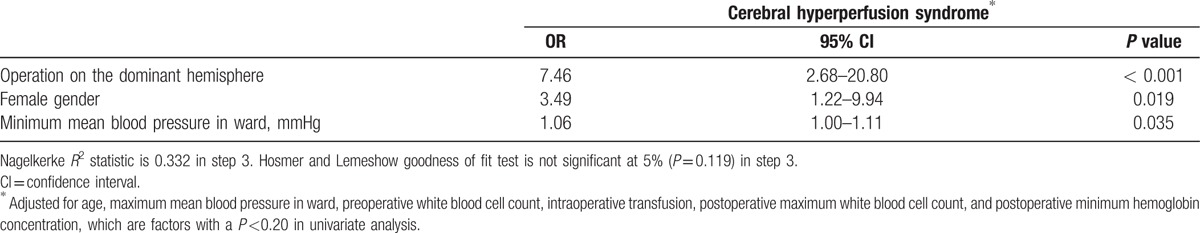
Independent predictors for cerebral hyperperfusion syndrome after superficial temporal artery-middle cerebral artery anastomosis in adult patients with moyamoya disease on a forward stepwise binary logistic regression.

## 4 Discussion

This study found that the incidence of postoperative CHS was not affected by intraoperative dexmedetomidine administration. However, the duration of CHS was shorter in patients with moyamoya disease who received intraoperative dexmedetomidine compared with those not given intraoperative dexmedetomidine.

CHS, which is characterized by focal neurological deficits due to cerebral edema, unilateral headache, facial pain, seizures, and intracranial hemorrhage, is a major postoperative complication after direct revascularization surgery in moyamoya disease, with a reported incidence of 17% to 50%.^[2,3,8]^ Although the pathogenesis of CHS is not clear, a rapid increase in cerebral blood flow in the chronic ischemic area has been suggested to be mechanisms.^[3,8]^ In moyamoya disease, breakdown of the blood–brain barrier,^[9]^ impaired cerebral autoregulation^[10]^ can induce vasogenic cerebral edema by CBF increase without reactive cerebral vasoconstriction (autoregulation), which were regarded as major causes. Increased vascular permeability and oxygen-derived free radical also have been considered other causes. In patients with moyamoya disease, up-regulation of matrix metalloproteinase-9, which is a proteolytic enzyme degrading vascular structure, can increase vascular permeability^[11]^ Also, elevated interleukin-1 may be related to cerebral vasodilation and hyperemia after vascular reperfusion in moyamoya patients.^[12,13]^ Moreover, in ischemia/reperfusion injury, oxygen-derived free radicals can damage vascular endothelium, resulting in impaired cerebral autoregulation.^[14,15]^ Treatment strategies include strict blood pressure control, the prevention of intracranial hemorrhage, and the administration of free oxygen radical scavengers.^[16]^

Dexmedetomidine, a selective α_2_-agonist, has shown neuroprotective effects in various experimental animals.^[17,18]^ Although the exact mechanism remains unknown, dexmedetomidine has many beneficial biochemical properties, such as antioxidant effects,^[19]^ anti-inflammatory effects,^[20,21]^ suppressed glutamate release,^[22]^ and apoptosis regulation.^[23]^ Moreover, it preserves the regional CBF and produces an optimal balance in the microregional oxygen supply and consumption during severe hemorrhagic hypotension.^[5]^ It also improves the microregional oxygen balance during reperfusion after focal cerebral ischemia.^[4]^ With respect to postoperative CHS, a previous study demonstrated that dexmedetomidine was effective in treating postoperative CHS in patients undergoing carotid endarterectomy.^[7]^ Hence, we expected that it would also have beneficial effects on postoperative CHS in patients with moyamoya disease after direct revascularization surgery.

However, intraoperative dexmedetomidine did not reduce the incidence of postoperative CHS. We postulate that the indications for surgery and duration of dexmedetomidine administration contributed to this result. The surgical indications for direct revascularization in patients with moyamoya disease may depend on the experience and training of the neurosurgeon.^[1]^ At our institution, moyamoya patients with repetitive, progressive symptoms, in particular symptoms and signs associated with the dominant hemisphere of the brain, and significant hemodynamic instability proven by perfusion imaging such as acetazolamide-challenge SPECT or perfusion MRI are indications for MCA-STA anastomosis. In other words, because of the strict surgical indications, most patients likely had a long preoperative period of cerebral ischemia/infarction, which resulted in long-term perfusion limitations causing maximal vasodilation of microvessels in the affected area. The beneficial effects of intraoperative dexmedetomidine on postoperative CHS might be abrogated because such microvascular dilation can hinder dexmedetomidine-induced vasoconstriction. In addition to the surgical timing, the short duration of intraoperative dexmedetomidine administration might contribute to our result. In this study, most patients received dexmedetomidine infusion after STA-MCA anastomosis and it was stopped just before ICU transfer because we had no appropriate protocol for the use of dexmedetomidine in postoperative care for patients with moyamoya disease. In contrast, in previous studies that have reported positive effects of dexmedetomidine in patients who underwent cardiac surgery, dexmedetomidine was administered continuously during the surgical procedure and during the postoperative period in the ICU.^[24,25]^ The small amount of dexmedetomidine administered during surgery may be related to our results because the neuroprotective effect of dexmedetomidine has been regarded to be dose-dependent.^[26]^ Therefore, we believe that continuous administration of dexmedetomidine during postoperative period in the ICU might affect our main result, the incidence of CHS.

Interestingly, this study showed that the duration of CHS and length of hospital stay were shorter in patients who received intraoperative dexmedetomidine. In both experimental and clinical studies, dexmedetomidine can decrease CBF,^[5]^ and such a CBF reduction may be related to the decrease of cerebral metabolic activity due to its sedative effect.^[27]^ Dexmedetomidine also showed an anti-inflammatory effect.^[21]^ Dexmedetomidine inhibited the production of inflammatory cytokines such as interleukin-6, interleukin-8, and tumor necrosis factor-alpha.^[28]^ Such proinflammatory cytokines-induced vasodilation and hyperemia are considered another cause of CHS.^[29]^ In addition, dexmedetomidine showed antioxidant effect by inhibiting lipid peroxidation process.^[29]^ The levels of malodialdehyde, a product of lipid peroxidation, and hypoxanthine, which promote reactive oxygen species production, were significantly decreased under dexmedetomidine infusion in cases with anticipated ischemia/reperfusion injury on upper extremities.^[29]^ Taken together, although our result did not support the protective effect of dexmedetomidine on postoperative CHS, we believe that the use of intraoperative dexmedetomidine may at least affect the clinical course of postoperative CHS.

There were several limitations to this study. First, this was a retrospective study conducted in a single center with small sample size, which may affect the ability to detect significant findings in some instances. Second, in the present study, since intraoperative administration of dexmedetomidine for preventing postoperative CHS in moyamoya patients is off-label use, there was a possibility of a bias in patient selection, despite a similarity in demographics between 2 groups. Moreover, the protocol for dexmedetomidine administration was uniform. The total dose and duration of dexmedetomidine administered may affect the incidence of postoperative CHS. Therefore, various studies on the dose of dexmedetomidine and timing and duration of dexmedetomidine administration are needed. Third, in the present study, the diagnosis of CHS was mainly based on clinical data. Because cerebral blood flow study such as SPECT was not always performed in all cases to confirm the diagnosis of CHS, a caution is needed in interpreting our results about the incidence of CHS.

In conclusion, intraoperative dexmedetomidine administration did not reduce the incidence of postoperative CHS in patients who had undergone direct revascularization surgery for moyamoya disease, although it reduced the duration of CHS. A further prospective study is necessary to thoroughly evaluate the effects of intraoperative dexmedetomidine on postoperative CHS.
